# Smoking behaviours among pregnant women in the Baffin Region of Nunavut

**DOI:** 10.3402/ijch.v72i0.21894

**Published:** 2013-07-31

**Authors:** Chantal R. M. Nelson

**Affiliations:** Institute of Population Health, University of Ottawa, Ottawa, Canada

The thesis examined three different research questions to help build a knowledge base for future intervention strategies by better understanding the reasons behind smoking among pregnant women in Nunavut. The first study focused more at the individual level and investigates clinical and socio-economic factors and their relationship to the readiness to quit smoking. The second article moved beyond the individual level to the broader social and structural environment to identify a broader range of barriers and facilitators to smoking and smoking cessation among Inuit women. This second article draws upon in-depth interviews focusing on perceptions of smoking, and perceived barriers and facilitators of smoking behaviours. Finally, the third article investigated the perspectives of health care providers regarding the barriers and facilitators of smoking cessation for pregnant women in the Baffin Region of Nunavut and describes perceptions of smoking cessation resources available to health care providers in the this Region. It is hoped that the findings from this thesis will help generate a knowledge base that can more appropriately address the smoking cessation needs of Inuit pregnant women.

